# Scrutinizing the Profile and Risk Factors of Suicide: A Perspective from a Case–Control Study Focused on a Northern Region of Spain

**DOI:** 10.3390/ijerph192315867

**Published:** 2022-11-29

**Authors:** María Sáenz-Aldea, María T. Zarrabeitia, Ana García Blanco, Ana Santurtún

**Affiliations:** 1 Family and Community Medicine, Davila Health Center, Health Service of Cantabria, Unit of Legal Medicine, University of Cantabria, 39005 Santander, Spain; 2 Unit of Legal Medicine, Department of Physiology and Pharmacology, University of Cantabria, IDIVAL, 39005 Santander, Spain; 3 Pathology Service, Institute of Legal Medicine of Cantabria, Unit of Legal Medicine, University of Cantabria, 39005 Santander, Spain

**Keywords:** suicide, risks factors, socio-demographic variables, cancer, chronic pain, psychiatric disease

## Abstract

Suicide is a major public health problem the prevention of which has become a priority, and, to this end, knowledge of its risk factors is essential. This study aims to evaluate the impact of some social, medico-legal, and clinical issues on suicide deaths. A total of 135 cases were identified as suicides that occurred in a region of northern Spain between 2018 and 2020. Controls (three for each case) were matched by age, sex, and urban–rural areas. The information was collected retrospectively through electronic health record systems. A binary logistic regression analysis was performed to study the association between individual risk factors and suicide. Being male (78.5%), between 40 and 60 years of age, unmarried (70.9%), and unemployed (85%) were associated with suicide deaths. Although the existence of a previous self-harm attempt is presented as the most robust risk factor (OR 22.121 [8.997–54.389]), the presence of a psychiatric diagnosis (OR 12.583 [7.686–20.601]) and cancer (OR 3.729 [1.845–7.536]) also showed a significant relationship with suicide (*p* < 0.05). Defining and knowing the risk factors for suicide helps to better understand the profiles of those individuals who are vulnerable, and enables prevention actions to be taken in both social and medical spheres.

## 1. Introduction

Suicide is considered a serious public health problem, as it represents one of the leading causes of unnatural death. The World Health Organization (WHO) reported more than 700,000 suicides during 2019 worldwide, accounting for up to 1.3% of the total deaths [1]. In Europe, an average of 11 suicides per 100,000 inhabitants was estimated in 2017 [2]. In Spain, despite having one of the lowest suicide mortality rates in Europe, the number of deaths by suicide is increasing annually according to the National Institute of Statistics (INE) [3]. In addition to all this, there is the problem of documenting suicide deaths, which are thought to be underreported by up to 10–30% according to some series [4,5].

Suicide is the result of a complex interaction between environmental [6,7], socio-economic [8,9], biological [10], and psychological factors [11].

The WHO published that between 10–15% of people who have attempted suicide, died by suicide, and indicated that the risk of death in people who have attempted self-harm is 100 times higher than in the general population [12]. There are many risk factors associated with suicide, some of which are a background of trauma during childhood [13,14], psychiatric comorbidity (particularly depression) [15,16], and other diseases (usually linked to disability or chronic pain) [17].

Some studies have evaluated the roles of different socio-demographic variables on suicide [18]. Overall, death by suicide is more frequent in older men, while suicide attempts have been described with a higher frequency in women [19,20]. On the other hand, the transition from work to retirement could act as a triggering factor [21,22].

Defining and knowing the risk factors for suicide helps us to better understand the profiles of vulnerable individuals and enables prevention actions to be undertaken from the social and medical spheres. It also facilitates the development of early detection programs in which all social and health professionals can take part [23,24].

The aim of this study is to analyze the socio-demographic characteristics and medical variables (psychiatric and somatic diseases) of people who died by suicide in order to get a perspective of the associated factors in the northern part of Spain.

## 2. Materials and Methods

The present study was conceived as a retrospective, analytical, observational epidemiological study with a case-control design. The study was carried out in the Autonomous Community of Cantabria, a region located in northern Spain with a population of approximately 580,000 inhabitants. To address the main objective of the study, the clinical profiles of individuals who died by suicide was reviewed and compared with people taken from the general population, using individuals selected from 3 different Primary Care Centers.

### 2.1. Selection of Cases and Controls

For the creation of the sample, both groups were defined, and the inclusion and exclusion criteria were established. The selection of the cases was based on the autopsies provided by the Institute of Legal Medicine of Cantabria.

The case group consisted of people whose medico-legal etiology of death was categorized as suicide in Cantabria between January 2018 and December 2020. The resulting sample size was 135 cases, of which, 11 did not have sufficient data for identification, or whose clinical history was not available because they were foreigners or from another region.

**Figure 1:**
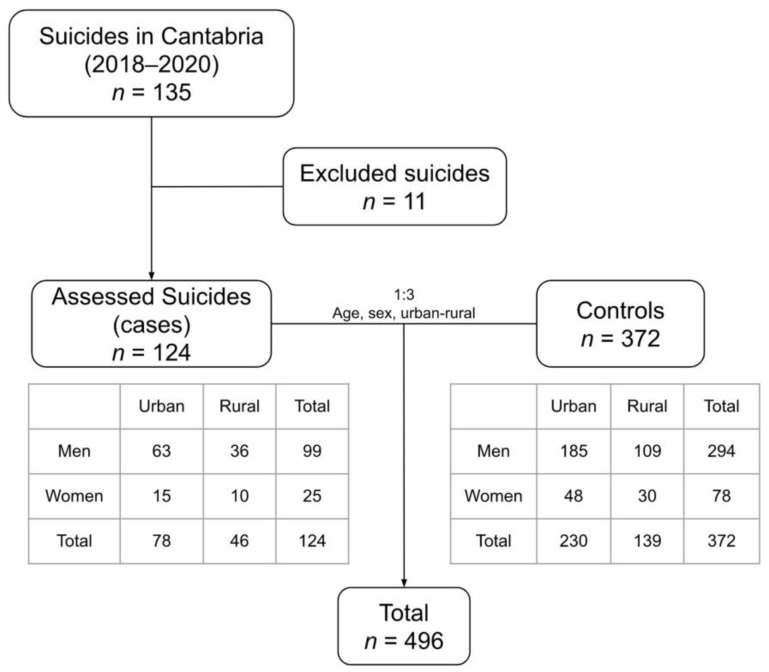
Diagram of study subject selection.

The control group consisted of all people who were alive at the time of the study, and who were registered in the databases of the Cantabrian Health Service (SCS). Three controls were selected for each case, matching them by sex, age, and urban–rural area. Therefore, the final sample was 496 people: 124 cases and 372 controls (Figure 1).

The final sample for the case-control analysis consisted of 393 men (79%: 99 cases and 294 controls) and 103 women (21%: 25 cases and 78 controls) with a ratio of 3.8:1. The mean age was 57.7 years and the median 56.5 years, with a mean age in men of 59.1 years and in women of 52.7 years.

### 2.2. Data Collection

The electronic health records of Primary Care and Hospital Care were used for data collection once the characteristics of the patients were confirmed and their suitability for inclusion in the study was verified.

For the descriptive analysis of suicides deaths, the following variables were collected: sex, age, marital status, employment status, nationality, place of residence, suicide method, place where the self-inflicted act took place, history of abuse, and previous self-harm attempts.

A total of 25 variables related to physical and psychiatric health were also collected for the case-control study.

### 2.3. Data Analysis

In order to evaluate the association between the socio-demographic variables and suicide, we used the Z test for the comparison of proportions. We used data obtained from the INE and the Cantabrian Institute of Statistics (ICANE), and the Spanish Institute of Statistics (INE) population during the first quarter of the year 2019 as a reference sample since the data of interest were absent from many of the controls’ medical histories. The chi-square test was applied to evaluate the differences between sexes.

For the case–control study, Pearson’s chi-square test and Fisher’s exact test were used to contrast proportions, and a binary logistic regression model was built with those independent variables that reached statistical significance (*p* < 0.05) in the univariate analysis.

### 2.4. Ethical Aspects

The study was based on routine clinical practice, with no alteration to normal patient care. It was approved by the Research Ethics Committee with Medical Products of Cantabria (CEIm of Cantabria) and complies with the Regulation (EU) 2016/679 of the European parliament and of the Council of 27 April 2016 on the Protection of natural persons with regard to the processing of personal data and on the free movement of such data and with the Spanish Organic Law 3/2018 of December 5 on the Protection of Personal Data and Guarantee of Digital Rights [25]. The study investigators have guaranteed the confidentiality of the patients included in the study.

## 3. Results

In Cantabria, 135 suicides took place between 2018 and 2020, of which 106 (78.5%) were males and 29 (21.5%) were females. The mean age was 57.2 years, being 58.7 years for males and 51.7 years for females. The age distribution showed a predominance of suicides in the 40–59 age group with 40% of suicides occurring in this age group. The next most frequent age group was between 60 and 79 years of age (30%). Suicides were more infrequent in people aged 39 years or less (18%) and 80 years or more (12%).

### 3.1. Social Factors

**Figure 2:**
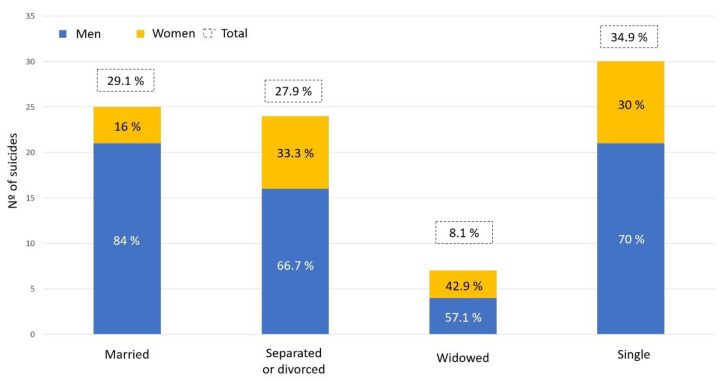
Graphical representation of the marital status of the study sample.

In total, 34.9% of the people who died by suicide were single, 29.1% were married, 27.9% were divorced, and 8.1% were widowed. Compared with the marital status of the general Spanish population, there is a higher proportion of divorced people in our sample and a lower percentage of married people, see Table 1. These differences are statistically significant in both cases (*p* < 0.0001) (Figure 2).

**Figure 3:**
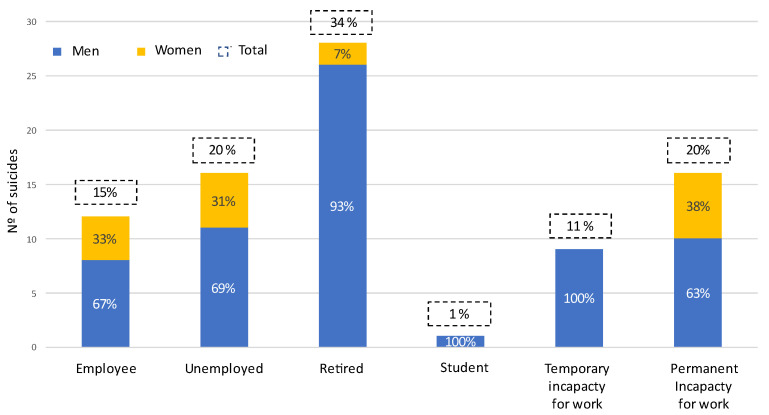
Graphical representation of the employment situation of the study people.

In terms of employment, 15% of the people who died by suicide were employed and active, while the highest percentage were retired (34%), and 20% had a permanent disability. Considering the situation in relation to work activity assessed by sex, it should be noted that while most men were retired (40%) and the most frequent situation among women was having a permanent disability (35.3%) (Figure 3). The differences between sexes are considerable although not statistically significant (*p* = 0.08).

### 3.2. Residence Characteristics

In the sample studied, 94% of the people had a Spanish nationality, while 6% were foreigners. Taking Spanish residents as a reference, the proportion of the Spanish population that died by suicide was statistically higher than that of the foreign population (*p* = 0.0097), see Table 1.

Living alone is identified as a risk factor for suicide. Out of all those who died by suicide, 38.8% lived alone (37.9% for men, 40.9% for women) with statistically significant differences from the reference population for both sexes (*p* < 0.0001), see Table 1.

According to the place of residence, 63.2% of the people in the study lived in an urban environment (>10,000 inhabitants), and the remaining 36.8% of the sample lived in a rural environment. This distribution is similar to the national distribution (*p* = 0.795), see Table 1.

### 3.3. Medico-Legal Features

Hanging was the most frequently used suicide method, representing 40% of the sample (45.3% of men and 20.7% of women). In women, jumping from a high place was the most-used method with a frequency of 27.6%. Firearms were exclusively used by the males in the sample (11.3% of the males). Taking into account, urban–rural differences, hanging was the most widely used method overall (33% in urban areas and 52% in rural areas). On the other hand, jumping from a high place was more prevalent in urban (29%) than in rural areas (11%).

Regarding the place where the intentional self-harm act took place, 53.6% were committed in the home and 35.7% in a public place. In total, 10% of the people were reported to be in an institution at the time of the suicide (prison, hospital, nursing home, etc.).

Examining the history of abuse, 8% of the patients in the sample had suffered abuse during their childhood (29% physical abuse, 29% emotional neglect, 21% sexual abuse and 7% physical neglect, while the remaining 14% had suffered abuse, but the type of abuse was not specified in their medical records). In relation to abuse in adulthood, 4% of the clinical histories reviewed reported having suffered abuse, all of them women. Of this latter group, 67% had suffered physical abuse and 33% psychological.

### 3.4. Medical History

Univariate regression analysis shows that the most robust risk factors for death by suicide were the presence of a previous self-harm attempts, which was present in 27% of the cases with an OR of 22.121 (95% CI 8.997–54.389); and the existence of a diagnosis of previous psychiatric pathology, which was present in two-thirds of the cases (67%) with an OR of 10.740 (95% CI 6.738–17.118), see Table 2.

The results of the Binary logistic regression model when including only somatic variables show that cancer (OR 2.021; 95% CI 1.105–3.696, *p* = 0.022) and chronic pain under opioid treatment (OR 2.201; 95% CI 1.055–4.976, *p* = 0.046) were significantly associated with suicide.

When taking into account the diagnosis of a psychiatric disorder, only mental conditions (OR 12.583; 95% CI 7.686–20.601) and cancer (OR 3.729; 95% CI 1.845–7.536) were significantly associated with suicide (*p* < 0.001), while chronic pain under opioid treatment was not (*p* = 0.06).

## 4. Discussion

This work is the first case-control study carried out in Spain that aims to define the clinical profile of suicides. In addition, it attempts to explore the relationship of suicide with variables not well-represented in the literature, such as somatic diseases.

Suicide affects the population differently depending on their sociodemographic characteristics. We have observed that living alone, being separated or divorced, and being unemployed are more prevalent among people dying by suicide than in the general population. These results coincide with those of other researchers in terms of gender distribution [26,27,28]. Also, the incidence is markedly higher in men (specifically, in our sample the ratio is 3.8 men for every woman), which is in line with previous studies and with similar ratios given the region where the study took place. Moreover, the highest number of suicides was concentrated in people between 45 and 60 years of age, which correlates with the data from the European population [29].

Suicide occurred more frequently among the separated or divorced and less frequently in the married group, as has also been described by other researchers [30,31]. However, the differences between rural and urban settings found in other studies do not appear in our sample [32].

Hanging is the most commonly used suicide method worldwide, which is in line with our findings; however, in the sample studied, jumping from a high place was more common than firearm-related deaths, findings that do not match with other studies [33]. It should be taken into account that Spain has strict firearm regulation laws, which has been correlated to a decreased percentage of suicide deaths by this method in other European countries [34].

A history of maltreatment in childhood was found in 8% of the cases studied, but it is likely that the real percentage is higher, as this information is not routinely gathered when establishing a clinical history. Santa Mina and Gallop defined childhood trauma as a risk factor for suicidal behavior [13], and Molnar et al. reported the relationship between childhood sexual abuse and suicidal behavior [14].

In our results, the two variables most strongly associated with suicide were the presence of psychiatric pathology and previous self-harm attempts. Psychiatric illnesses are associated with a higher risk of premature mortality from both natural and unnatural causes [35]. The literature has described an increased risk in practically all pathologies but especially in depression, schizophrenia, and alcohol and other substance abuse [36,37]. In addition, the presence of a previous history of suicide attempt should initiate alert protocols and serve as a “red flag” for the detection of potentially vulnerable people towards whom to direct personalized attention should be directed [38,39,40].

In the case of diseases associated with cardiovascular risk factors, such as hypertension, diabetes mellitus (DM), and dyslipidemia, none of these showed statistically significant differences between the people who had died by suicide and the control group. Although there are studies in the literature that show that the populations with DM [41] or dyslipidemia [42] have a higher number of suicide attempts and a greater risk of suicide, other studies have not found the same association [43].

Likewise, statistical significance was also not reached for respiratory, cardiac, and renal failure. Some authors have described an association between suicide and asthma, COPD, heart failure, and renal failure [43,44]. Nonetheless, most of the studies on somatic pathologies focus more on suicidal ideation than on suicide, so more literature is needed to clarify these relationships.

Rheumatologic diseases and patients with HIV infection were not as strongly represented in our study groups, so statistical significance was not reached in any of them. Studies focused on inflammatory pathology seem to describe an increased risk of suicide in people with SLE [45], a finding that we have not been able to corroborate given the lack presence of people with SLE in our sample. In the case of HIV infection, studies have linked it to suicide in the presence of other psychiatric comorbidities [46].

In line with our results, some authors have described that cancer is a risk factor for suicide [47] and have even hypothesized that there may be an underestimation of suicide deaths here, as they may have been wrongly categorized as accidental or unintentional deaths due to medication misadministration [48].

In our study, chronic pain was considered to be pain whose diagnosis was recorded as such in electronic health records. We also took into account those patients who were under follow-up by the Pain Unit or the Musculoskeletal Unit, or those who had repeated consultations for refractory pain. Besides this, we wanted to distinguish between chronic pain with and without opioid treatment for several reasons: the first was to avoid possible confounding biases, and the second was because those patients treated with opioids probably suffer pain of greater intensity or that is more refractory to treatment, and which is therefore, experience more severe pain. Proof of this is that in our study, the statistical significance of chronic pain under treatment with opioids was reached, thus being a risk factor for suicide.

There are several studies that highlight the importance of pain as a potentially independent risk factor for suicide [49,50,51,52], and our findings reinforce the need for targeted support and intervention efforts for people with chronic pain.

Neurological diseases are a major cause of disability. The diagnosis of a neurodegenerative disease is often accompanied by physical and psychological pain and hopelessness. There is a higher prevalence of suicide in patients diagnosed with a neurological pathology, and especially in those diagnosed with neurodegenerative pathology than in the general population [53]. In the case of our study, we were unable to demonstrate this relationship in any of the neurological items: stroke, spinal cord injury, neurodegenerative diseases, dementia, Parkinson’s disease, Alzheimer’s disease, Huntington’s disease, or MS. This is probably due to the scarcity of cases with neurological disease in our sample, so more studies with a larger sample size are needed in order to explore this association.

### Limitations

Suicide is a multifactorial phenomenon, and our results allow us to develop a better understanding of this cause of death, but they cannot predict suicidal behavior; we are defining correlation but not causation. The retrospective nature of the study is a limitation related to the heterogeneity of the data collection, which was carried out by different doctors and does not always detail the analyzed variables. Some variables could not be included in the 135 participants despite having reviewed the different electronic medical files (both in the health centers and in the hospitals in the region). Moreover, some of the studied variables showed a very low incidence which resulted in weakening the power of our analyses.

## 5. Conclusions

Defining and knowing the risk factors for suicide helps us to better understand the profiles of those who are vulnerable and enables preventative actions to be taken in both social and medical spheres.

The results of this study show that not all deaths by suicide are the exclusive result of psychiatric pathology alone. Therefore, prevention strategies should also focus on socio-economic risk factors and medical comorbidities.

Being male, between 40 and 60 years of age, unmarried, and unemployed were the most frequent contexts in individuals that died by suicide. The existence of a previous self-harm attempt is presented as the most robust risk factor, followed by the presence of a psychiatric diagnosis, cancer, and severe chronic pain. Given the high prevalence of many of these factors, it is important to screen for suicidal ideation or behavior on a regular basis in daily clinical practice.

## Figures and Tables

**Table 1:** Comparison of proportions between cases in our sample and the Spanish population (according to INE’s data).

Variables	Sample (%)	Spanish Population (%)	Z-Score	*p*	CI 95%
Divorced	27.9	6.5	7.84	<0.0001	(0.114–0.315)
Married	29.1	52.5	4.2	<0.0001	(−0.334–−0.130)
Spanish nationality	94.3	85.3	2.59	0.0097	(0.041–0.132)
Living alone	38.8	10.3	8.2	<0.0001	(0.172–0.398)
Urban environment	63.2	64.7	0.26	0.795	(−0.104–0.073)
Rural environment	36.8	35.3	0.26	0.795	(−0.073–0.104)

**Table 2:** Distribution of study variables and results of univariate analysis using Chi-square and Fisher’s test.

Variables	Cases		Controls		*p*	OR (IC 95%)
	*n*	%	*n*	%		
Psychiatric diagnosis	83	67%	59	16%	<0.001	10.740 (6.738–17.118)
High blood pressure	43	35%	137	37%	0.666	0.911 (0.595–1.394)
Dyslipidemia	42	34%	117	31%	0.617	1.116 (0.725–1.719)
Previous self-harm attempt	33	27%	6	2%	<0.001	22.121 (8.997–54.389)
Cancer	20	16%	32	9%	0.018	2.043 (1.121–3.724)
Diabetes Mellitus	17	14%	51	14%	1	1 (0.554–1.806)
Heart Failure	15	12%	26	7%	0.074	1.831 (0.936–3.582)
Rheumatological disease	13	10%	21	6%	0.065	1.958 (0.949–4.037)
Chronic pain treated with opioids	12	10%	17	5%	0.036	2.237 (1.037–4.827)
Chronic pain treated without opioids	11	9%	23	6%	0.309	1.477 (0.698–3.124)
COPD	8	6%	11	3%	0.102	2.263 (0.889–5. 762)
Renal Insufficiency	8	6%	11	3%	0.102	2.263 (0.889–5.762)
Stroke	8	6%	16	4%	0.334	1.534 (0.640–3.678)
OSA	7	6%	27	7%	0.538	0.764 (0.324–1.802)
Asthma	6	5%	21	6%	0.732	0.850 (0.335–2.156)
Neurogenerative disease	5	4%	10	3%	0.544	1.521 (0.510–4.539)
Dementia	3	2%	10	3%	0.871	0.898 (0.243–3.315)
HIV infection	3	2%	6	2%	0.697	1.512 (0.373–6.140)
Parkinson disease	3	2%	4	1%	0.374	2.281 (0.503–10. 335)
Fibromyalgia	2	2%	1	0%	0.156	6.082 (0.547–67.659)
Spinal cord injury	2	2%	4	1%	0.643	1.508 (0.273–8. 336)
Huntington disease	1	1%	0	0%	0.25	0.248 (0.213–0.290)
SLE	0	0%	1	0%	1	0.749 (0.712–0.789)
Alzheimer disease	0	0%	3	1%	0.577	0.748 (0.711–0.788)
MS	0	0%	2	1%	0.413	0.749 (0.712–0.788)

COPD: Chronic Obstructive Pulmonary Disease; OSA: Sleep Apnea-Hypopnea Syndrome; HIV: Human Immunodeficiency Virus; SLE: Systemic Lupus Erythematosus; MS: Multiple Sclerosis.

## Data Availability

The data that support the findings of this study are available from the corresponding author upon reasonable request.
